# SMITER—A Python Library for the Simulation of LC-MS/MS Experiments

**DOI:** 10.3390/genes12030396

**Published:** 2021-03-11

**Authors:** Manuel Kösters, Johannes Leufken, Sebastian A. Leidel

**Affiliations:** Department of Chemistry, Biochemistry and Pharmaceutical Sciences (DCBP), University of Bern, Freiestrasse 3, 3012 Bern, Switzerland; manuel.koesters@dcb.unibe.ch (M.K.); johannes.leufken@dcb.unibe.ch (J.L.)

**Keywords:** mass spectrometry, simulation, gold standard, nucleosides, proteomics, benchmarking

## Abstract

SMITER (Synthetic mzML writer) is a Python-based command-line tool designed to simulate liquid-chromatography-coupled tandem mass spectrometry LC-MS/MS runs. It enables the simulation of any biomolecule amenable to mass spectrometry (MS) since all calculations are based on chemical formulas. SMITER features a modular design, allowing for an easy implementation of different noise and fragmentation models. By default, SMITER uses an established noise model and offers several methods for peptide fragmentation, and two models for nucleoside fragmentation and one for lipid fragmentation. Due to the rich Python ecosystem, other modules, e.g., for retention time (RT) prediction, can easily be implemented for the tailored simulation of any molecule of choice. This facilitates the generation of defined gold-standard LC-MS/MS datasets for any type of experiment. Such gold standards, where the ground truth is known, are required in computational mass spectrometry to test new algorithms and to improve parameters of existing ones. Similarly, gold-standard datasets can be used to evaluate analytical challenges, e.g., by predicting co-elution and co-fragmentation of molecules. As these challenges hinder the detection or quantification of co-eluents, a comprehensive simulation can identify and thus, prevent such difficulties before performing actual MS experiments. SMITER allows the creation of such datasets easily, fast, and efficiently.

## 1. Introduction

Mass spectrometry (MS) has transformed our ability to systematically analyze biological systems. Even though mostly used in proteomics, modern mass spectrometers are capable of determining the mass and abundance of essentially any biomolecule at high resolution. This versatility has made MS indispensable in many research areas of the omics world, including proteomics, metabolomics, glycomics, and lipidomics, but also in modomics, the analysis of chemically modified RNA nucleotides [1,2,3,4,5]. Parallel to the development of faster and more sensitive instruments, wet-lab protocols have introduced new processing schemes, chromatographic protocols, and labeling methods to conduct highly sophisticated measurements. The increasing complexity of experimental designs has boosted the necessity to develop computational tools for, e.g., the analysis of biomedical MS data. Therefore, a plethora of software solutions have been published by labs from the different research fields [6,7,8]. All these tools are designed to address key challenges in computational mass spectrometry: (i) data are inherently noisy despite all attempts to generate pure samples; (ii) shadowing of signal by ion suppression; (iii) while being highly accurate, different types of measurement errors can influence the results; (iv) even state-of-the-art instruments are generally not fast enough to measure reliably all analytes contained within a sample; finally, (v) the variability of chromatographic separation renders results difficult to compare.

One of the key steps in any computational mass spectrometry pipeline is the annotation of molecular features that are measured in a liquid-chromatography-coupled tandem mass spectrometry (LC-MS/MS) run. This process is commonly called “peak-” or “feature detection” and is of particular importance when extracting quantitative information, since quantifications are more prone to instrument- and chromatography-dependent variations. Existing software solutions mainly focus on proteomics data as the most prominent biomedical application of modern mass spectrometry [9,10,11,12,13]. However, the rapid development of new instruments allows for the analysis of new classes of molecules. This has triggered a surge of interest in detecting and quantifying other biomolecules including small molecules like modified nucleotides [3,14] and has resulted in numerous new algorithms to perform the crucial step of feature detection. Importantly, the benchmarking and development of such algorithms is challenging, since annotated gold-standard datasets for metabolomics are difficult to generate. Such datasets that reflect the ground truth need to contain a defined composition of analytes as well as defined parameters like cycle- and exclusion time and the accuracy of the instrument during data acquisition. In general, gold-standard datasets are generated by the manual annotation of the measurements, which is a daunting task considering that hundreds of thousands of spectra can be generated in a single experiment. Even if such datasets exist, they are based on specific instrument settings and might not contain the features or information that are critical for novel types of analyses.

Faced with this challenge, the proteomics community has simulated gold-standard datasets using computational tools like MSSimulator [15], Mspire [16], or JAMSS [17]. These tools need to be able to simulate several key features in MS measurements like (i) retention time (RT), (ii) ionization, (iii) raw signals, (iv) isotope patterns, (v) isotope- or chemical labels, (vi) shapes of the elution profile, (vii) resolution, (viii) noise, (ix) ion-sampling rate, (x) exclusion time, (xi) fragmentation, and (xii) ion-injection time. Recently, specialized tools have also been developed for other areas like lipidomics [4]. Similar to the proteomics simulators, these tools are highly specific for one type of molecule. This specialization is surprising since a simulator can treat any molecule based on its mass that is defined by a chemical formula. Indeed, a generalized software solution for simulating MS data is still lacking.

Here, we present a new software solution that closes this gap. SMITER (Synthetic mzML writer) is a universal Python tool able to simulate MS data for any biomolecule. It uses an input file that consists of chemical formulas with corresponding RT and charge. Retention time and charge are essential features linked to an analyte in MS experiments. As such they need to be provided as an input to SMITER either based on real measurements or predicted by using established tools [18,19]. Based on the input molecules, SMITER uses pyQms to calculate highly accurate isotopic patterns [20], which are assembled to chromatographic features in the RT dimension. Subsequently, these features are scaled using a custom function. Gaussian-, γ-, and exponentially modified Gaussian distributions are currently implemented. Noise on the *m*/*z* and intensity levels is injected along the chromatographic features. Implemented are uniform noise and a noise model that combines general noise with intensity-specific noise similar to the model proposed in Mspire [16]. Finally, MS2 fragmentation scans including noise are generated using the fragmentation function of pyteomics [21] for peptides and the fragments as defined in the Modomics database [22,23,24] for modified nucleosides. To simulate lipidomics, we have implemented LipidCreator [25,26]. For each simulated LC-MS/MS run, SMITER generates an output file in mzML format. Therefore, SMITER offers new options to benchmark algorithms used to analyze MS data reaching far beyond the proteomics and modomics area, since modules for any biomolecule can easily be implemented.

## 2. Material and Methods

SMITER is written in Python (version 3.8) using best practices in software development such as (i) unit testing, (ii) integration testing, (iii) code documentation, (iv) type hints, and (v) example scripts. The program utilizes other libraries that are frequently used in computational mass spectrometry such as psims [27], pyQms [20], and common scientific Python libraries like numpy and scipy [28].

The retention time of each molecule is defined in the input .csv file. These retention times are either taken from real measurements or by using prediction tools. For each molecule, isotope envelopes are calculated using pyQms [20] and noise is added to the simulated value. MS1 and MS2 peaks are generated in the same manner; however, MS2 peak *m*/*z* values are generated by pyteomics [21] or another user-defined software.

### 2.1. Noise Calculation

The experimental noise of the chromatographic peak is essentially modeled as described in [16]. Briefly, the noise is drawn from a normal distribution, where the standard deviation depends on the intensity values. Noise values are calculated using:

(1) $$\sigma =m\left(1-e^{-ci}\right)+d$$

where *i* is the intensity of the molecule, *c* controls the increase in sigma with increasing intensity, *d* is the minimum sigma, and *m* + *d* the maximum sigma, and

(2) $$\sigma =mi^{-y}$$

where *m* is the maximum sigma, *i* is the intensity, and *y* is an experimentally derived constant determining the decrease in sigma with increasing intensities as described in [16]. Following noise injection, all intensities are normalized to the maximum intensity within each spectrum prior to chromatographic peak scaling.

### 2.2. MS1 Peak Generation

pyQms (running with default parameters) is used to generate MS1 isotopic envelopes for a given molecule [20]. For each molecule and charge state, the *m*/*z* values for all isotopes of the envelope are precalculated and saved for fast access. The generation of *m*/*z* values can be adjusted using pyQms parameters, e.g., by adding a fixed machine offset (MACHINE_OFFSET_IN_PPM). Following feature calculations, all features are scaled by applying a user-defined model. By default, we have implemented an exponentially modified Gaussian distribution. Alternatively, Gaussian or γ distributions can be used.

### 2.3. MS2 Peak Generation

For each MS1 spectrum, all ions are selected in a user-defined window centered around the top N peaks, and fragment ions are calculated by using pyteomics [21] or peptide_fragments (GitHub: https://github.com/fufezan-lab/peptide_fragments: accessed on 10 March 2021). Molecules are only fragmented if they match the simulation parameters for, e.g., dynamic exclusion and minimum intensity. Subsequently, noise is injected using the same model as in the MS1 scan. After generation of all fragment *m*/*z* values, their corresponding intensities are scaled using the same strategy as for the parent ion. Additionally, the user can specify a fragmentation efficiency for each molecule, which is multiplied with the scaled intensity.

### 2.4. Chromatographic Feature Assembly

In order to generate the chromatographic features, each isotope envelope in an MS1 spectrum needs to be scaled according to its own peak shape function and parameters. Since the molecules in one MS1 spectrum elute at different timepoints relative to the peak function, each envelope is scaled separately in an iterative process. The difference between two spectra is set by the user. The default is 50 ms, resulting in a cycle time of 1 s for a single MS1 scan and 20 MS scans.

### 2.5. Simulation of RNA Nucleosides

An LC-MS/MS run of RNA nucleosides was simulated based on a complex mix of nucleoside standards defined in Sarin et al. [29]. A gradient length of 80 min was used and nucleoside reference RTs were taken from a reference LC-MS/MS run of the described complex standard mix. Fragment *m*/*z* values were implemented using the Modomics database [22,23,24]. The scan time difference was set to 60 ms. For generating *m*/*z* noise, m was set to 0.001701 and y to 0.2 [16]. A total white noise of 1000 distributed over a random number of peaks (between 1 and 100) was introduced according to the section “Noise calculation”. Fragments were generated using the custom fragment library from MORAST (Leufken et al., manuscript in preparation) and 10% of all fragment ions in a given scan were randomly omitted.

### 2.6. Simulation of Proteomics Sample

An example proteomics run was simulated based on a reference run from Bruderer et al. [30]. Peptides and their corresponding RTs are based on the identifications of the reference run. To obtain identifications, the ursgal framework [31] was used to search the file using X!Tandem [32]. Precursor tolerance was set to 5 ppm and fragment tolerance to 0.02 Da. Carbamidomethylation of cysteine was set as a fixed modification; oxidation of methionine and acetylation of the N-termini of proteins were used as optional modifications. The results were validated using Percolator (v3.4.0) [33]. Peptide spectrum matches (PSMs) were filtered to a false-discovery rate (FDR) of 0.01. Subsequently, an input file for SMITER was generated to simulate a 140 min gradient, similar to the measurement published in [30]. A scan time difference of 60 ms was used. Noise was introduced similar to the simulation of RNA nucleosides. Theoretical peptide fragments were generated using pyteomics [21].

### 2.7. Benchmarking of Proteomics Sample

A benchmark dataset was generated by simulating a tryptic digest of the full proteome of *Saccharomyces cerevisiae* and sampling sets of peptides of different sizes. RTs were predicted using deeplc and peak width was drawn from a uniform distribution between 30 and 120 s. Dynamic exclusion was set to 30 s, minimum intensity was set to 500, and a maximum of 20 fragment spectra per MS1 spectrum was allowed. The charge state was drawn from a multinominal distribution and peak intensity from a uniform distribution between 100,000 and 1,000,000. The simulated data were searched using X!Tandem [32] with a precursor mass tolerance of 5 ppm and a fragment mass tolerance of 0.02 Da. The search results were validated using Percolator (v3.4.0) [33] and filtered to a q-value of 1%. After this, the numbers of peptide spectrum matches (PSMs) and unique peptide sequences were counted.

### 2.8. Code Access

The full code, example scripts, and additional information can be found online in the public GitHub repository: (github.com/LeidelLab/SMITER: accessed on 10 March 2021).

## 3. Results

We sought to develop a Python tool to simulate LC-MS/MS runs of RNA nucleosides to optimize and to benchmark software tools for the analysis of chemical RNA modifications. However, to benefit from the versatility of mass spectrometry, we chose to develop a universal tool that can similarly be used for the simulation of proteomics experiments and in other areas of modern mass spectrometry.

**Figure 1:**
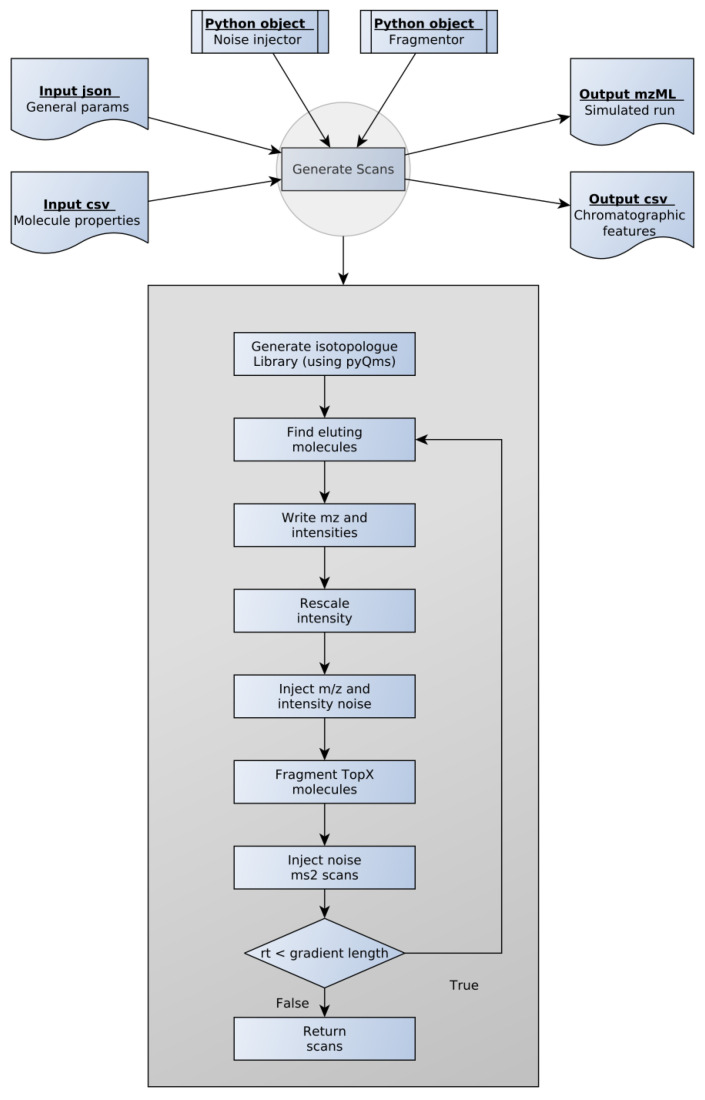
Overview of the SMITER workflow. For each simulation, the user provides two input files containing the general parameters and the properties of the molecules. Two output files are generated. The simulated run is provided in mzML format while the chromatographic features are written in csv format. Each file type is indicated as a blue box. The key steps of scan generation are shown in the grey box.

Hence, we designed SMITER (Figure 1). The program requires two main input files. First, general run parameters such as gradient length and minimum intensity as well as the parameters for generating isotope traces through pyQms are defined in a .json file. Second, the analytes and their properties such as, e.g., peak apex, are defined in a .csv file. To facilitate the use of SMITER, we provide an example script for generating an input .csv file based on a FASTA file using RT prediction through deeplc [18]. A second example uses published RTs of RNA nucleosides derived from the Modomics database [22,23,24]. Both can be found on the GitHub page of SMITER (github.com/LeidelLab/SMITER: accessed on 10 March 2021). However, for other biomolecules, different prediction algorithms or databases can be implemented according to the preferences of the users.

To generate the individual scans, SMITER requires two Python objects: First, a noise-injector object to implement the different types of noise that are anticipated in real measurements. Second, a fragmentation object to define the fragments that are expected during MS2 fragmentation. For noise injection or for the definition of the molecular fragmentation pattern, users can use the default settings of SMITER or implement their preferred tool. As default, SMITER uses the MS-spire noise model [16] and the fragmentation model defined by pyteomics [21]. Finally, pyQms [20] is used to calculate accurate isotope patterns, which are written as peaks into the spectra.

SMITER calculates spectra as long as the RT of the most current spectrum is smaller than the defined gradient length using a user-defined spectrum RT difference. The default of 50 ms can be adjusted based on the assumed instrument performance. At each retention time, all molecules eluting at this timepoint are retrieved from an interval tree. Subsequently, *m*/*z* values of the corresponding isotopes are retrieved from the precalculated library and intensities are rescaled according to the user-defined distribution. By default, an exponentially modified Gaussian distribution is used, since this distribution has been shown to be the best model for chromatographic elutions [34,35,36]. However, alternative options such as a normal Gaussian distribution or a γ distribution can be chosen by the user. Next, noise is injected as described in the Material and Methods. Whenever required by the user, random white noise can also be added to the spectra. SMITER generates MS2 fragment spectra for the N most intense peaks as defined by the user. In essence, fragments of all molecules within a defined *m*/*z* window are calculated or retrieved from the specified source. Similarly, noise is injected and intensities are rescaled using the same distribution as for the parent ion. The user can define exclusion time to simulate measurements more realistically. Finally, the output is written into a mzML file using psims [27] and an overview .csv file is created to store all simulated features.

**Figure 2:**
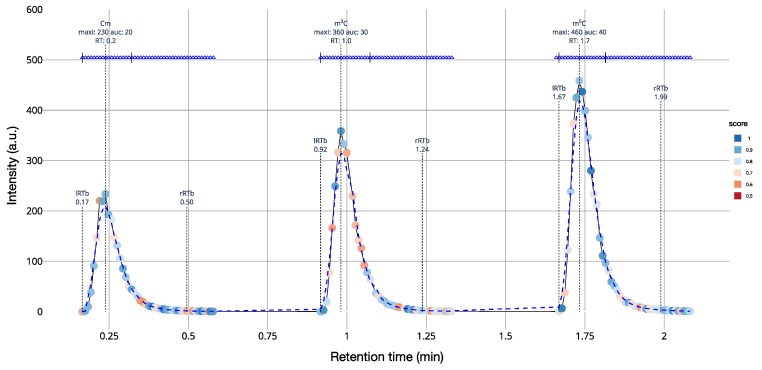
Extracted ion chromatograms from a simulated liquid-chromatography-coupled tandem mass spectrometry (LC-MS/MS) run containing the modified cytidines: 2′-*O*-methylcytidine (Cm), 3-methylcytidine (m^3^C), and 5-methylcytidine (m^5^C) analyzed and visualized by MORAST. The color of the dots represents the matching accuracy of the isotope envelopes, with a score of 1 indicating a perfect match (dark blue), and a score of 0.5 indicating a weak match (dark red). Color gradient was set up using ColorBrewer (colorbrewer2.org: accessed on 10 March 2021). Blue triangles at the top mark spectra, for which the molecule was identified in the corresponding MS2 spectrum.

As an example, we generated an extracted ion chromatogram of 2′-*O*-methylcytidine (Cm), 3-methylcytidine (m^3^C), and 5-methylcytidine m^5^C (Figure 2). These three isomeric nucleosides cannot be distinguished based on their *m*/*z* value and require MS2 fragmentation to be unambiguously identified. Our simulation includes *m*/*z* values and intensity noise. Identifications that are based on MS2 spectra are indicated as blue triangles. The quality of the match between the theoretical and the simulated isotope pattern is calculated by pyQms and indicated by color. In this representation, perfect matches are indicated by dark blue (score = 1), while weak matches are indicated by dark red (score = 0.5; Figure 2). Nucleosides were quantified and identified using MORAST (Modified RNA Analysis Search Tool), a software for the analysis of high-resolution bottom-up MS data of modified RNA nucleosides (Leufken et al., manuscript in preparation).

**Figure 3:**
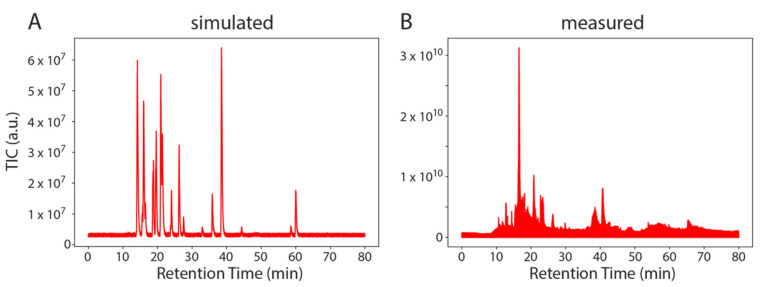
Example of SMITER output. Two total ion chromatograms (TIC): simulated (**A**) and measured (**B**) from a mix of modified nucleosides. Intensity is plotted on the Y-axis (a.u.) over the retention time (min).

Next, we used SMITER to simulate an entire LC-MS/MS run of synthetic modified nucleosides that contained 24 nucleoside standards similar to measurements performed in [29] (Figure 3). The direct comparison between the simulated and the real measurement shows that the simulated run exhibits lower noise levels. This is expected since the simulated run contains exclusively the specified target molecules, while nucleoside standards contain an undefined set of contaminants such as byproducts of the chemical synthesis but also ions that are not nucleosides. It is important to note that the most intense peak in the measured run (at a RT of approximately 15 min) is not observed in the simulated run. This peak is an unidentified contamination and as such, has not been simulated. The remaining peaks appear comparable. Finally, a basic white noise level is evident both in the measured sample as well as in the simulated MS run.

**Figure 4:**
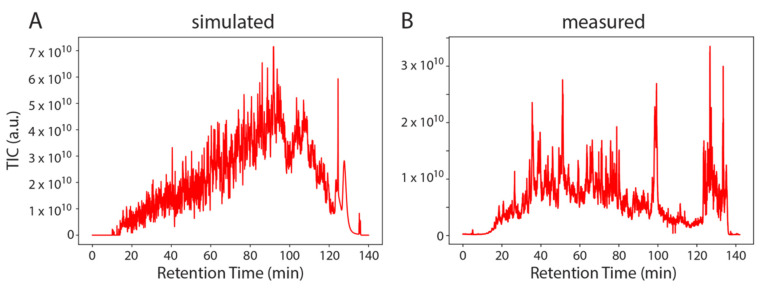
Example of SMITER output. Two total ion chromatograms (TIC): simulated (**A**) and measured (**B**) from a complex mix of yeast peptides. Intensity is plotted on the Y-axis (a.u.) over the retention time (min).

To demonstrate the universality of SMITER, we applied the same procedure to a complete proteomics experiment where we simulated the peptides identified in one LC-MS/MS run of the Bruderer et al. dataset [30] (Figure 4). In general, the simulated run shows more distinct features than the measurement. Similar to the simulation of modified RNA nucleosides, the simulated run lacks non-peptide contaminants, which are known to elute after approximately 120–140 min in real LS-MS/MS experiments.

The simulated run shows constantly increasing total ion chromatograms (TIC) with no major intensity drops for a longer RT window, while, on the other hand, the measurement does not constantly increase and shows a larger variation. Furthermore, the proteases used for the generation of peptides do not work efficiently on all substrates. Hence, tryptic peptides will vary more than in an in silico digest. Finally, not all yeast proteins are expressed in exponentially growing yeast cells. Therefore, the simulated peptides do not fully represent the ex vivo sample. However, the user can simulate these features more realistically by adapting the FASTA file. The simulated run can be used to efficiently test different labeling strategies, since the simulation can use any label, without performing an actual experiment. This allows the simulation of differential labeling prior to conducting an experiment. In addition to simulating data based on identifications of an actual experiment, SMITER can also be used to simulate experiments without previous identifications, based on RT predictions of peptides using, e.g., deeplc [18].

SMITER allows the user to quickly adapt the simulated experimental conditions. The simulation of a yeast proteome with a gradient length of 140 min, 36,854 peptides, and 11,640 MS2 scans using a top10 method takes less than three minutes on a standard laptop. If desired, more elaborated simulations, tools to predict ionization, or charge states can be easily incorporated into the workflow.

**Figure 5:**
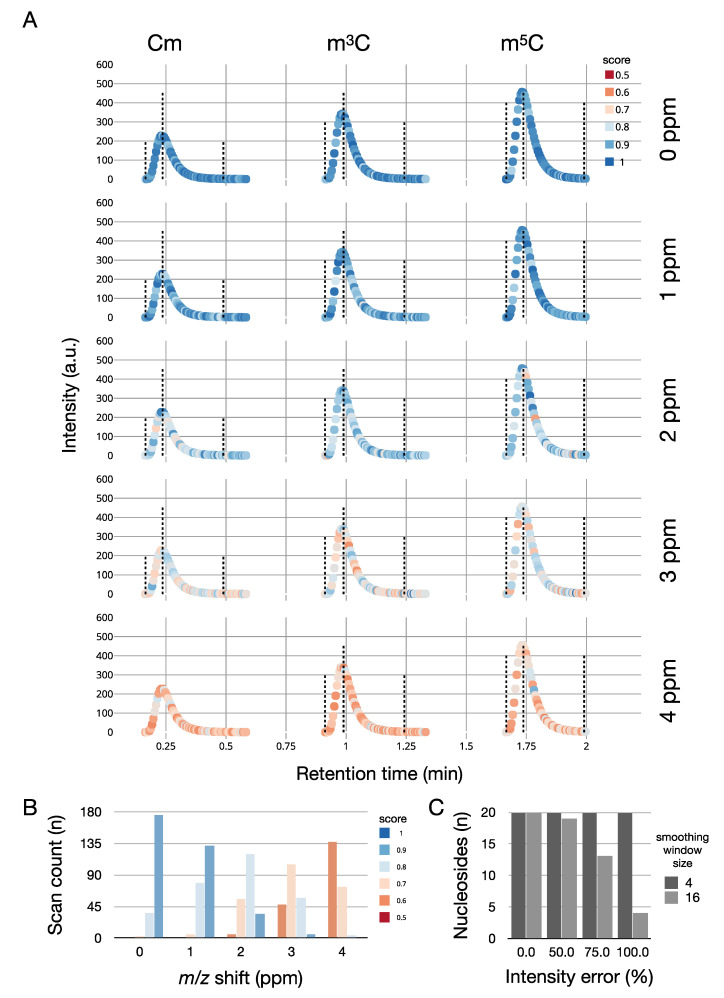
(**A**) Simulation of different ppm errors for the three nucleosides 2′-*O*-methylcytidine (Cm), 3-methylcytidine (m^3^C), and 5-methylcytidine m^5^C. Absolute intensities are plotted on the Y-axis, retention time on the x-axis. The quality of the mass spectrum is indicated by color: dark blue (high quality), dark red (low quality). The dotted lines indicate the start of the peak, the peak apex, and the end of the peak (from left to right). (**B**) Number of spectra with a pyQms-quantification score for all methylated cytidines above a certain threshold, dependent on the introduced *m*/*z* shift error in ppm. (**C**) Number of detected nucleosides dependent on the introduced intensity error. Values are shown before (smoothing window size = 16, light grey) and after (smoothing window size = 4, dark grey) parameter optimization.

To show an example of how SMITER can be used to benchmark MS software, we repeated the simulation of Cm, m^3^C, and m^5^C. However, this time, we systematically varied the ppm offset of the mass spectrometer from 0 to 4 ppm and analyzed the data using MORAST (Figure 5A). Without ppm errors, the quantifications yield optimal results. However, the introduction of less precise measurements leads to a decrease in quantification quality reflected in a high number of spectra with low scores for the isotope pattern matches. (Figure 5B). We further evaluated SMITER for optimizing parameters of MORAST in simulated experiments. Introduction of different levels of intensity noise led to a decreased detection of nucleosides in a simulated run due to the failure of the peak-detection routine to determine peaks correctly. By adjusting the parameter that influences the smoothing of the raw data prior to peak detection, all chromatographic peaks could be detected (Figure 5C).

**Figure 6:**
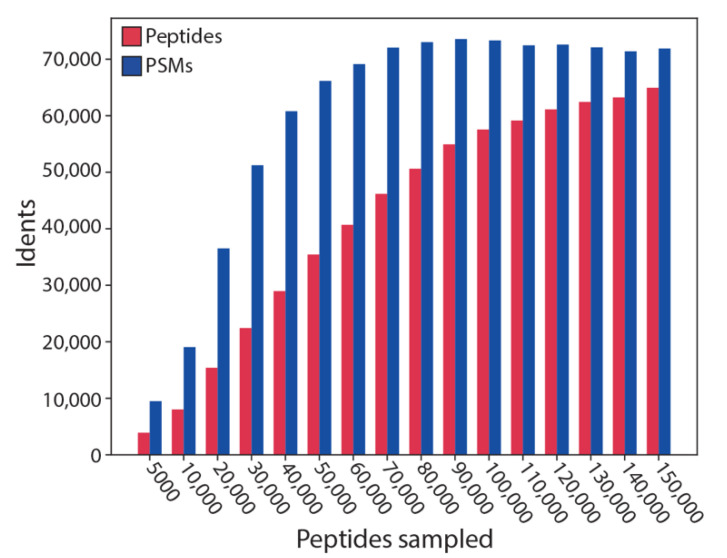
Performance test of SMITER simulation. The tryptic peptides of a full yeast proteome were simulated; different numbers of peptides were randomly sampled and analyzed using X!Tandem and Percolator. The number of identified peptide spectrum matches (PSM) and of identified peptides are indicated in response to the number of sampled peptides.

Finally, in order to test the performance of SMITER, we assessed how many peptides of a simulation can be identified using an established peptide database search engine. Hence, we simulated peptide sets of different sizes, searched the data with X!Tandem followed by a verification using Percolator (Figure 6). Interestingly, for small peptide sets, almost all peptides were identified (90% for 5000), most of them through multiple PSMs. With a growing number of simulated peptides, the identification rate is reduced to less than 50% for >130,000 simulated peptides. This reflects that the chromatographic run becomes increasingly saturated by peptides that overlap during elution. Therefore, the mass spectrometer is unable to trigger MS2 identifications for those ions since they are excluded based on a data-dependent acquisition (DDA) top 20 method. This, however, is a reflection of a true effect that is also observed in real MS experiments, where, e.g., a fractionation leads to an increase in peptide identifications. These results show that SMITER is, indeed, able to simulate realistic MS experiments.

## 4. Discussion

Here, we presented SMITER, a new and efficient tool to simulate LC-MS/MS experiments. SMITER bases all its calculations on the chemical formula of the analyte and is, therefore, not limited to a specific class of molecules or labeling technique. Following this strategy, our software enables the simulation of MS data in any field of metabolomics, lipidomics, and modomics, where universal and efficient simulation tools are lacking. Due to the implementation in Python, SMITER is a versatile tool that can utilize a rich infrastructure of program modules and can be easily integrated into existing workflows. Alternatively, new workflows can be rapidly prototyped using SMITER.

Analysis software for mass spectrometry constantly needs to adapt to new protocols and instruments. Whenever a new application is developed, this requires testing with defined samples that match the new experimental pipeline. The generation of hand-annotated gold-standard datasets is labor intensive, time-consuming, and therefore, expensive. The simulation of LC-MS/MS runs offers a cheap, fast, and efficient strategy to optimize algorithms and to pinpoint potential limits of planned analyses. Finally, simulated runs can help to evaluate a labeling strategy or experimental setup before conducting expensive and time-consuming experiments. Hence, SMITER can act as a base for the improvement and integration of new and existing tools in computational mass spectrometry, in particular when the amounts of the analyte are limited. However, each analyte elutes at a specific time point during a chromatographic gradient. Therefore, the elution time of each molecule has to be provided to SMITER, either by using actual measurements or from prediction tools. This limitation could theoretically be solved by randomly assigning elution times. However, such an assignment would have little to do with a real sample and has, therefore, not been implemented.

We have used SMITER to test and to improve the peak detection routine of our analysis software MORAST, a tool for the automated analysis of nucleosides in bottom-up high-resolution MS data. For example, simulating different peak–apex distances revealed an unexpected behavior in the routine that MORAST uses to assign MS2 spectra to the related chromatographic peaks. Isomers appear with identical *m*/*z* values in the MS data and cannot be distinguished without additional information such as the elution order or MS2 identification. If two isomers elute in close proximity, the task becomes even more challenging since the MS2 spectra will contain an unknown composition of two different molecules with the same sum formula and identical *m*/*z* values. A simulated reference dataset can help to detect and to resolve issues in the program code such as the mis-assignment of chimeric MS2 spectra, how to adjust the weighting of fragment peaks in MS2 spectra, peaks that fail to be assigned, or other unexpected errors. This can help to overcome a common challenge in modomics and metabolomics, as frequently, isomers need to be resolved reliably [29].

Since important parameters such as peak shape or the isolation window can be defined, potential issues like co-fragmentation of ions can be evaluated before performing cost- and time-consuming experiments. We have evaluated this on a simulated dataset for 74,067 peptides with overlapping simulated and measured retention times. We found 13,830 chimeric spectra based on two molecules that were selected for fragmentation at the same time. A total of 2032 spectra showed fragments of three or more peptides. The highest number of peptides that were fragmented within a single spectrum was nine, a scenario that occurred twice. However, SMITER requires an input of elution times of the analytes to perform its predictions.

Currently, SMITER users can adjust 21 parameters that can vary in MS experiments. It will depend on the demand of the community which features will be adapted and implemented in the future. Our software is open source and freely available on GitHub. Users can commit and contribute to the development of SMITER and, for example, introduce fragmentation algorithms, RT predictors, or features that are characteristic for specific classes of molecules. SMITER has the strong potential to develop through interactions with the growing MS community. Therefore, we invite everybody to contribute.
